# New Stabilization for Dynamical System with Two Additive Time-Varying Delays

**DOI:** 10.1155/2014/315817

**Published:** 2014-02-18

**Authors:** Lianglin Xiong, Fan Yang, Xiaozhou Chen

**Affiliations:** School of Mathematics and Computer Science, Yunnan University of Nationalities, Kunming 650500, China

## Abstract

This paper provides a new delay-dependent stabilization criterion for systems with two additive time-varying delays. The novel functional is constructed, a tighter upper bound of the derivative of the Lyapunov functional is obtained. These results have advantages over some existing ones because the combination of the delay decomposition technique and the reciprocally convex approach. Two examples are provided to demonstrate the less conservatism and effectiveness of the results in this paper.

## 1. Introduction

As is well known, delay systems are frequently encountered in various practical systems, such as engineering systems, biology, economics, and neural networks [1–8]. So, the past decades have witnessed extensive research on time delays in the literature including stability analysis, stabilization H*∞* controllers design, robust filtering analysis, and model reduction or simplification [9–15].

Recently, a new model of system with two additive time-varying delay components was proposed in [16]. This model has a strong application background in remote control and networked control. Take a state-feedback networked control, for example. Since the physical plant, controller, sensor, and actuator are located at different places, signals are transmitted from one device to another. Thus time delays will appear. Among those delays there are two network-induced ones, one from sensor to controller and the other from controller to actuator. With the two delays considered, the closed-loop system will appear with two additive time delays in the state. Because of the network transmission conditions, the two delays are generally time-varying with different properties. Therefore it is of significance to consider stability for systems with two additive time-varying delay components. The stability analysis was addressed in [16], and a delay-dependent stability criterion was obtained; it was further improved in [17], where a marginally delayed state was exploited to construct Lyapunov functional and more free weighting matrices were introduced to estimate the upper bound of the derivative of the Lyapunov functional. However, that leaves much room for improvement on the stability criteria in [16, 17]. In [18], a new Lyapunov functional is constructed to derive new condition for the two additive time-varying delay systems. This paper makes full use of and any useful terms in the calculation of the time derivative. It is seen that *d*_1_(*t*), *d*(*t*) − *d*_1_(*t*),  and *h* − *d*(*t*) are not simply enlarged as *h*_1_, *h* − *h*_1_, and *h*, respectively. Instead, the relationship of *d*_1_(*t*) + (*h*_1_ − *d*_1_(*t*)) = *h*_1_, (*h* − *d*(*t*)) + (*d*(*t*) − *d*_1_(*t*)) − (*h*_1_ − *d*_1_(*t*)) = *h* − *h*_1_, and *d*(*t*) + (*h* − *d*(*t*)) = *h* is considered. The stability conditions are in form of many linear matrix inequalities in this paper. However, many LMIs caused by a integral representation may bring conservative. Reference [19] presents a less conservative result for stability analysis of continuous-time systems with additive delay by constructing a new Lyapunov-Krasovskii functional and utilizing free matrix variables in approximating certain integral quadratic terms in obtaining the stability condition in terms of linear matrix inequalities. However, it is easy to see that the stability criteria for the given delay bound ${\bar{\tau }}_{2}$ are good while for the given delay bound ${\bar{\tau }}_{1}$ are undesirable, compared to some other recent paper. The stabilization for systems with two additive time-varying delays is studied by [20]. Different from [17], the terms *d*_1_(*t*)*N*(*Z*_1_+*Z*_2_)^−1^ *N* ^*T*^, (*h*_1_ − *d*_1_(*t*))*TZ*_1_^−1^ *T* ^*T*^, *d*_2_(*t*)*MZ*_2_^−1^ *M* ^*T*^, and (*h* − *d*(*t*))*SZ*_2_^−1^ *S* ^*T*^ are not enlarged roughly but kept as they are and handled using the convex polyhedron method. This paper is less conservative than [16, 17]. However, similar to [18], many LMIs may also cause conservativeness. Combining with a reciprocally convex combination technique, the new stability condition is obtained [21]. Different from [16, 17, 19], the information about *d*(*t*), *d*_1_(*t*), and *d*_2_(*t*) is fully considered in the constructed Lyapunov functional. So far, [21] has presented the best results for delay-dependent stability analysis for delay systems with two additive time-varying delays. In fact, the results provided by [21] are also conservative to some extent, which motivates the study of this paper.

In this paper, the problem of stabilization analysis for continuous-time systems with two additive time-varying delay components is investigated. Firstly, with the idea of delay decomposition, by considering the independence and the variation of two additive time-varying delay components, a new class of Lyapunov functionals is constructed. Combining with a tighter estimation of the derivative of the Lyapunov functional and a reciprocally convex combination technique [22], new delay-dependent stability criteria with less conservatism are derived in terms of linear matrix inequalities (LMIs). Secondly, based on the obtained stability conditions, with the new introduced positive scalar, the controller is designed for the control systems. Finally, two numerical examples are also given to show the effectiveness and the improvement of the proposed method.

*Notation.* Throughout this paper, a real symmetric matrix *P* > 0  (≥0) denotes *P* as being a positive definite (positive semidefinite) matrix. *I* is used to denote an identity matrix with proper dimension. Matrices, if not explicitly stated, are assumed to have compatible dimensions. The symmetric terms in a symmetric matrix are denoted by ★.

## 2. Problem Statement

Consider the following time-delay system with two additive time-varying delays:

(1) $$\begin{array}{c} \dot{x}\left(t\right)=Ax\left(t\right)+Bx\left(t-d_{1}\left(t\right)-d_{2}\left(t\right)\right)+Du\left(t\right),\\ x\left(t\right)=\phi \left(t\right),\;t\in \left[-d,0\right], \end{array}$$

where *x*(*t*) ∈ *R* ^*n*^ is the state vector, *u*(*t*) ∈ *R* ^*m*^ is the control input which is arranged as *u*(*t*) = *Kx*(*t*). *A* ∈ *R* ^*n*×*n*^, *B* ∈ *R* ^*n*×*n*^, *D* ∈ *R* ^*n*×*m*^, and *K* ∈ *R* ^*m*×*n*^ are constant matrices, and *ϕ*(*t*) is the initial condition function. The time delays *d*_1_(*t*) and *d*_2_(*t*) are time-varying differentiable functions that satisfy

(2) $$\begin{array}{c} 0\leq d_{1}\left(t\right)\leq d_{1},\;\;0\leq d_{2}\left(t\right)\leq d_{2}, \end{array}$$

(3) $$\begin{array}{c} 0\leq {\dot{d}}_{1}\left(t\right)\leq \mu_{1}<\infty ,\;\;0\leq {\dot{d}}_{2}\left(t\right)\leq \mu_{2}<\infty , \end{array}$$

where *d*_1_, *d*_2_ and *μ*_1_, *μ*_2_ are constants. Naturally, we denote

(4) $$\begin{array}{c} d\left(t\right)=d_{1}\left(t\right)+d_{2}\left(t\right),\;\;d=d_{1}+d_{2},\;\;\mu =\mu_{1}+\mu_{2}. \end{array}$$

In fact, system (1) belongs to a special class of systems with single delay:

(5) $$\begin{array}{c} \dot{x}\left(t\right)=Ax\left(t\right)+Bx\left(t-d\left(t\right)\right)+Du\left(t\right), \end{array}$$

where *d*(*t*) satisfies 0 ≤ *d*(*t*) ≤ *d* and $\dot{\tau }(t)\leq \mu$. And when *D* = 0, the system (5) becomes

(6) $$\begin{array}{c} \dot{x}\left(t\right)=Ax\left(t\right)+Bx\left(t-d\left(t\right)\right). \end{array}$$

Our purpose of this paper is to study the stabilization for system (1), and the stability of (6) is firstly studied.

To end this section, we introduce the following lemmas, which will play an important role in the proof of the main results.

**Lemma 1 (see [2])**

For any constant matrix *M* ∈ *R* ^*n*×*n*^, *M* = *M* ^*T*^ > 0, and scalar *γ* > 0, the vector function *ω* : [−*r*,0] → *R* ^*n*^ such that the integrations concerned are well defined; then

(7) γ∫−γ0ωT(β)Mω(β)dβ≥(∫−γ0ω(β)dβ)T ×M(∫−γ0ω(β)dβ).

**Lemma 2 (see [22])**

Let *f*_1_, *f*_2_,…, *f*_*N*_ : *R* ^*m*^ ↦ *R* have positive values in an open subset **D** of  **R** ^*m*^. Then, the reciprocally convex combination of *f*_*i*_ over **D** satisfies

(8) $$\begin{array}{c} \underset{\left\{\alpha_{i}\;|\;\alpha_{i}>0,\;\sum_{i}\alpha_{i}=1\right\}}{\min}\sum_{i}\frac{1}{\alpha_{i}}f_{i}\left(t\right)=\sum_{i}f_{i}\left(t\right)+\underset{g_{i,j}\left(t\right)}{\max}\sum_{i\neq j}g_{i,j}\left(t\right) \end{array}$$

subject to

(9) $$\begin{array}{c} \left\{g_{i,j}:R^{m}⟼R,g_{j,i}\left(t\right)\equiv g_{i,j}\left(t\right),\;\left(\begin{array}{cc} f_{i}\left(t\right) & g_{i,j}\left(t\right)\\ g_{i,j}\left(t\right) & f_{i}\left(t\right) \end{array}\right)\geq 0\right\}. \end{array}$$

Our purpose of this paper is to study the stabilization for system (1), and the stability of (6) is firstly studied.

## 3. Main Results

### 3.1. Stability Analysis

**Theorem 3**

System (6) with delays *d*_1_(*t*) and *d*_2_(*t*) satisfying (2) and (3) is asymptotically stable if there exist matrices *P* = *P* ^*T*^ > 0, *Z*_*i*_ = *Z*_*i*_^*T*^ > 0  (*i* = 1,2, 3),  *W*_*i*_ = *W*_*i*_^*T*^ > 0  (*i* = 1,2,3), *Q*_11_,*Q*_12_,*Q*_22_, *R*_11_,*R*_12_,*R*_22_, *S*_*ij*_  (*j* ≥ *i*, *i* = 1,2,3,4, *j* ≤ 4), and *T*_12_,*T*_13_,*T*_23_ such that

(10) $$\begin{array}{c} \Sigma =\left(\begin{array}{cc} \Phi & \Psi \\ ★ & -M \end{array}\right)<0, \end{array}$$

(11) $$\begin{array}{c} S=\left(\begin{array}{cccc} S_{11} & S_{12} & S_{13} & S_{14}\\ S_{12}^{T} & S_{22} & S_{23} & S_{24}\\ S_{13}^{T} & S_{23}^{T} & S_{33} & S_{34}\\ S_{14}^{T} & S_{24}^{T} & S_{34}^{T} & S_{44} \end{array}\right)>0, \end{array}$$

(12) $$\begin{array}{c} \Gamma =\left(\begin{array}{ccc} W_{2} & T_{12} & T_{13}\\ T_{12}^{T} & W_{2} & T_{23}\\ T_{13}^{T} & T_{23}^{T} & W_{2} \end{array}\right)\geq 0, \end{array}$$

where Φ ∈ *R* ^12×12^ and Ψ ∈ *R* ^12×1^ are block matrices, such as

(13) Φ11=S11+S11−2W1−2W3−2W5 +Z1+Z2+Z3+PA+ATP,Φ12=S12+2W1,  Φ16=S14+2W3,Φ18=PB,  Φ1,10=S13+2W5,Ψ11=ATM,  Φ22=S22−4W1,Φ24=2W1,  Φ26=S24,Φ2,10=S23,  Φ33=−(1−μ1)Z1,Φ44=−2W1−W2,  Φ47=T12−T13,Φ48=W2−T12,  Φ49=T13,Φ55=−S22−2dW3d1−2dW3d2,  Φ56=2dW3d1−S12T,Φ59=2dW3d2−S24,  Φ5,12=−S23,Φ66=S44−S11−2W3−2dW3d1,  Φ69=−S14,Φ6,10=S34T,  Φ6,12=−S13,Φ77=T23−2∗W2+T23T+(u1−1)Z3,Φ78=W2−T12T+T13T−T23T,  Φ79=W2−T23,Φ88=T12−2W2+T12T+(u−1)Z2,  Φ89=T23−T13,Ψ8,1=BTM,  Φ99=−S44−W2−2W4−2dW3d2,Φ9,12=2W4−S34T,  Φ10,10=S33−4W5,Φ10,11=2W5,  Φ11,11=−2W4−2W5,Φ11,12=2W4,  Φ12,12=−S33−4W4,M=d12W1+d22W2+d2W3+d12W4+d22W5,

and the rest of the items of (10) are all zero.

**Proof**

Construct a Lyapunov functional candidate as

(14) $$\begin{array}{c} V\left(x\left(t\right)\right)=\sum_{i=1}^{6}V_{i}\left(x\left(t\right)\right), \end{array}$$

where

(15) V1(x(t))=xT(t)Px(t),V2(x(t))=∫t−d1(t)txT(s)Z1x(s)ds +∫t−d(t)txT(s)Z2x(s)ds +∫t−d2−d1(t)txT(s)Z3x(s)ds,V3(x(t))=∫t−d/2t(x(s)x(s−d12)x(s−d22)x(s−d2))T×S(x(s)x(s−d12)x(s−d22)x(s−d2))ds,V4(x(t))=∫−d10∫t+θtx˙T(s)d1W1x˙(s)ds dθ +∫−d−d1∫t+θtx˙T(s)d2W2x˙(s)ds dθ +∫−d0∫t+θtx˙T(s)dW3x˙(s)ds dθ +∫−d−d2∫t+θtx˙T(s)d1W4x˙(s)ds dθ +∫−d20∫t+θtx˙T(s)d2W5x˙(s)ds dθ,

*P*,*Z*_1_,*Z*_2_,*Z*_3_,*W*_1_,*W*_2_,*W*_3_,*W*_4_,*W*_5_, and *S*_*ij*_  (*j* ≥ *i*, *i* = 1,2,3,4, *j* ≤ 4) are matrices with appropriate dimensions to be determined.

The time derivative of *V*(*x*(*t*)) along the trajectory of system (6) is given by

(16) V˙1(x(t))=2xT(t)Px˙(t)=xT(t)(PA+ATP)x(t) +2xT(t)PBx(t−d(t)),

(17) V˙2(x(t))=xT(t)(Z1+Z2+Z3)x(t) −(1−μ1)xT(t−d1(t)) ×Z1x(t−d1(t)) −(1−μ)xT(t−d(t)) ×Z2x(t−d(t)) −(1−μ1)xT(t−d2−d1(t)) ×Z3x(t−d2−d1(t)),

(18) V˙3(x(t))=(x(t)x(t−d12)x(t−d22)x(t−d2))TS(x(t)x(t−d12)x(t−d22)x(t−d2)) −(x(t−d2)x(t−d+d12)x(t−d+d22)x(t−d))T ×S(x(t−d2)x(t−d+d12)x(t−d+d22)x(t−d)),

(19) V˙4(x(t))=−∫t−d1tx˙T(s)d1W1x˙(s)ds−∫t−dt−d1x˙T(s)d2W2x˙(s)ds−∫t−dtx˙T(s)dW3x˙(s)ds  −∫t−dt−d2x˙T(s)d1W4x˙(s)ds−∫t−d2tx˙T(s)d2W5x˙(s)ds+x˙T(t)Mx˙(t).

With the delay-partitioning approach and by Lemma 1, one can obtain that

(20) −∫t−d1tx˙T(s)d1W1x˙(s)ds  =−2∫t−d1/2tx˙T(s)d12W1x˙(s)ds   −2∫t−d1t−d1/2x˙T(s)d12W1x˙(s)ds  ≤−2(∫t−d1/2tx˙(s)ds)TW1(∫t−d1/2tx˙(s)ds)   −2(∫t−d1t−d1/2x˙(s)ds)TW1(∫t−d1t−d1/2x˙(s)ds)  =−2[x(t−d12)−x(t−d1)]T   ×W1[x(t−d12)−x(t−d1)]   −2[x(t)−x(t−d12)]TW1[x(t)−x(t−d12)],

(21) −∫t−dt−d1x˙T(s)d2W2x˙(s)ds  =−∫t−d(t)t−d1x˙T(s)d2W2x˙(s)ds   −∫t−d2−d1(t)t−d(t)x˙T(s)d2W2x˙(s)ds   −∫t−dt−d2−d1(t)x˙T(s)d2W2x˙(s)ds.

Now one can note that

(22) $$\begin{array}{c} \frac{d\left(t\right)-d_{1}}{d_{2}}+\frac{d_{2}-d_{2}\left(t\right)}{d_{2}}+\frac{d_{2}-d_{2}\left(t\right)}{d_{2}}=1. \end{array}$$

From Lemma 2 and (12) in Theorem 3, (21) can be given as follow

(23) −∫t−dt−d1x˙T(s)d2W2x˙(s)ds  ≤−(x(t−d1)−x(t−d(t))x(t−d(t))−x(t−d2−d1(t))x(t−d2−d1(t))−x(t−d))T   ×Γ×(x(t−d1)−x(t−d(t))x(t−d(t))−x(t−d2−d1(t))x(t−d2−d1(t))−x(t−d)).

Following a similar line as in the computation of $-\int_{t-d_{1}}^{t}{\dot{x}}^{T}(s)d_{1}W_{1}\dot{x}(s)ds$ in (20), we can obtain

(24) −∫t−dtx˙T(s)dW3x˙(s)ds=−2∫t−d/2tx˙T(s)d2W3x˙(s)ds−2∫t−(d+d1)/2t−d/2x˙T(s)dW3x˙(s)ds−2∫t−dt−(d+d1)/2x˙T(s)dW3x˙(s)ds  ≤−2[x(t)−x(t−d2)]TW3[x(t)−x(t−d2)]   −2dd1[x(t−d2)−x(t−d+d12)]T   ×W3×[x(t−d2)−x(t−d+d12)]   −2dd2[x(t−d+d12)−x(t−d)]T   ×W3×[x(t−d+d12)−x(t−d)],

(25) −∫t−dt−d2x˙T(s)d1W4x˙(s)ds  =−∫t−(d+d2)/2t−d2x˙T(s)d1W4x˙(s)ds   −2∫t−dt−(d+d2)/2x˙T(s)d1W4x˙(s)ds  ≤−2[x(t−d2)−x(t−d+d22)]T   ×W4×[x(t−d2)−x(t−d+d22)]   −2[x(t−d+d22)−x(t−d)]T   ×W4×[x(t−d+d22)−x(t−d)],

(26) −∫t−d2tx˙T(s)d2W5x˙(s)ds  =−2∫t−d2/2tx˙T(s)d22W5x˙(s)ds   −2∫t−d2t−d2/2x˙T(s)d22W5x˙(s)ds  ≤−2(∫t−d2/2tx˙(s)ds)TW5(∫t−d2/2tx˙(s)ds)   −2(∫t−d2t−d2/2x˙(s)ds)TW5(∫t−d2t−d2/2x˙(s)ds)  =−2[x(t−d22)−x(t−d2)]T   ×W5[x(t−d22)−x(t−d2)]   −2[x(t)−x(t−d22)]TW5[x(t)−x(t−d22)].

Hence, according to (16)–(26), we can obtain

(27) $$\begin{array}{c} \dot{V}\left(x\left(t\right)\right)\leq \xi^{T}\left(t\right)\Phi \xi \left(t\right)+{\dot{x}}^{T}\left(t\right)M\dot{x}\left(t\right), \end{array}$$

where

(28) ξT(t)=[xT(t)  xT(t−d12)  ×xT(t−d1(t))xT(t−d1)  ×xT(t−d+d12)xT(t−d2)  ×xT(t−d2−d1(t))xT(t−d(t))  ×xT(t−d)xT(t−d22)  ×xT(t−d2)xT(t−d+d22)],

(29) $$\begin{array}{c} M=d_{1}^{2}W_{1}+d_{2}^{2}W_{2}+d^{2}W_{3}+d_{1}^{2}W_{4}+d_{2}^{2}W_{5}. \end{array}$$

Inequality (27), which by the Schur complement on (10), is equivalent to

(30) $$\begin{array}{c} \dot{V}\left(x\left(t\right)\right)<0. \end{array}$$

Then we have $\dot{V}(x(t))<-\lambda |x^{2}(t)|$ for a sufficiently small positive constant *λ*, which means that system (6) with two additive time-varying delays *d*_1_(*t*) and *d*_2_(*t*) satisfying (2) and (3) is asymptotically stable. Accordingly, the proof is completed.

**Remark 4**

In this paper, the information about *d*(*t*), *d*_1_(*t*), *d*_2_(*t*),  and *d*_2_ + *d*_1_(*t*) is fully considered in Lyapunov functional (14). Not only that, but we also consider the information of *d*/2, *d*_1_/2, *d*_2_/2, (*d* + *d*_1_)/2,  and (*d* + *d*_2_)/2. So the Lyapunov functional in our paper is more general than the existing paper, and the stability criteria in our paper may be more applicable and less conservative.

**Remark 5**

It is noted that the delay decomposition is very important to improve the delay-dependent stability results. For example, this novel term *V*_3_(*x*(*t*)) is introduced in our Lyapunov functional. Furthermore, some techniques in the process of the computation for the derivative of the constructed Lyapunov functionals also play an important role in reducing conservativeness of our results. So, for example, $-\int_{t-d_{1}}^{t}{\dot{x}}^{T}(s)d_{1}W_{1}\dot{x}(s)ds$ is equivalently converted to two parts in (20), which is in concert with the derivative of the *V*_3_(*x*(*t*)), Because it would guarantee the quadratic term of *x*(*t* − *d*_1_/2) is negative in the derivative of *V*(*x*(*t*)) for the system. And there are similar settlements in other places.

**Remark 6**

The reciprocally convex combination lemma is important to increase the upper bound of time delay, although it brings many free matrices which may increase the complexity of the computation. It can be shown in the example. With the approach, it neither extends directly the *d*_1_(*t*) nor the *d*_2_(*t*) and *d*(*t*) to its upper bound or lower bound; thus, it would reduce the conservatism. In this paper, it just transfers the term $-\int_{t-d}^{t-d_{1}}{\dot{x}}^{T}(s)d_{2}W_{2}\dot{x}(s)ds$ to (23). It is worthwhile to note that convex polyhedral method may bring more conservativeness compared to the reciprocally convex method, because it always generates more than one conditions, which result in many matrices used again and again.

### 3.2. Controller Design

The approach of Theorem 3 can be used as a useful tool for the stabilization problem of system (1) with *u*(*t*) = *Kx*(*t*). Now we are in a position to design the controller by convex optimization approach.

**Theorem 7**

System (1) with *u*(*t*) = *Kx*(*t*) and two additive time-varying delays *d*_1_(*t*) and *d*_2_(*t*) satisfying (2) and (3) is asymptotically stable if there exist matrices $\bar{P}={\bar{P}}^{T}>0$,$\;{\bar{Z}}_{i}={\bar{Z}}_{i}^{T}>0\;\left(i=1,2,3\right)$,$\;{\bar{W}}_{i}={\bar{W}}_{i}^{T}>0\;(i=1,2,\ldots ,5)$, ${\bar{S}}_{ij}\;(j\geq i,\;i=1,2,3,4,\;j\leq 4)$, ${\bar{T}}_{12}$,${\bar{T}}_{13}$,${\bar{T}}_{23}$, and a scalar *λ* > 0, such that

(31) $$\begin{array}{c} \bar{\Sigma }=\left(\begin{array}{cccccc} \bar{\Phi } & Y & Y & Y & Y & Y\\ ★ & {\bar{\Omega }}_{1} & 0 & 0 & 0 & 0\\ ★ & ★ & {\bar{\Omega }}_{2} & 0 & 0 & 0\\ ★ & ★ & ★ & {\bar{\Omega }}_{3} & 0 & 0\\ ★ & ★ & ★ & ★ & {\bar{\Omega }}_{4} & 0\\ ★ & ★ & ★ & ★ & ★ & {\bar{\Omega }}_{5} \end{array}\right)<0, \end{array}$$

(32) $$\begin{array}{c} \bar{S}=\left(\begin{array}{cccc} {\bar{S}}_{11} & {\bar{S}}_{12} & {\bar{S}}_{13} & {\bar{S}}_{14}\\ {\bar{S}}_{12}^{T} & {\bar{S}}_{22} & {\bar{S}}_{23} & {\bar{S}}_{24}\\ {\bar{S}}_{13}^{T} & {\bar{S}}_{23}^{T} & {\bar{S}}_{33} & {\bar{S}}_{34}\\ {\bar{S}}_{14}^{T} & {\bar{S}}_{24}^{T} & {\bar{S}}_{34}^{T} & {\bar{S}}_{44} \end{array}\right)>0, \end{array}$$

(33) $$\begin{array}{c} \bar{\Gamma }=\left(\begin{array}{ccc} {\bar{W}}_{2} & {\bar{T}}_{12} & {\bar{T}}_{13}\\ {\bar{T}}_{12}^{T} & {\bar{W}}_{2} & {\bar{T}}_{23}\\ {\bar{T}}_{13}^{T} & {\bar{T}}_{23}^{T} & {\bar{W}}_{2} \end{array}\right)\geq 0, \end{array}$$

(34) $$\begin{array}{c} {\bar{\Omega }}_{1}=d_{1}^{-2}\left(\frac{1}{\lambda^{2}}{\bar{W}}_{1}-\frac{2}{\lambda }\bar{P}\right), \end{array}$$

where $\bar{\Phi }\in R^{12\times 12}$ and $\bar{\Psi }\in R^{12\times 1}$ are block matrices, such as

(35) Φ¯11=S¯11+S¯11−2W¯1−2W¯3−2W¯5+Z¯1 +Z¯2+Z¯3+LA+ATL+P¯A+ATP¯,Φ¯12=S¯12+2W¯1,  Φ16=S¯14+2W¯3,Φ¯18=BP¯,  Φ¯1,10=S¯13+2W¯5,Φ¯22=S¯22−4W¯1,  Φ¯24=2W¯1,Φ26=S¯24,  Φ2,10=S¯23,Φ¯33=−(1−μ1)Z¯1,  Φ¯44=−2W¯1−W¯2,Φ¯47=T¯12−T¯13,  Φ¯48=W¯2−T¯12,Φ¯49=T¯13,  Φ¯55=−S¯22−2dW¯3d1−2dW¯3d2,Φ¯56=2dW¯3d1−S¯12T,  Φ59=2dW¯3d2−S¯24,Φ¯5,12=−S¯23,  Φ¯66=S¯44−S¯11−2W¯3−2dW¯3d1,Φ¯69=−S¯14,  Φ¯6,10=S¯34T,Φ¯6,12=−S¯13,  Φ¯77=T¯23−2W¯2+T¯23T+(u1−1)Z¯3,Φ¯78=W¯2−T¯12T+T¯13T−T¯23T,  Φ¯79=W¯2−T¯23,Φ¯88=T¯12−2W¯2+T¯12T+(u−1)Z¯2,  Φ¯89=T¯23−T¯13,Φ¯99=−S¯44−W¯2−2W¯4−2dW¯3d2,  Φ¯9,12=2W¯4−S¯34T,Φ¯10,10=S¯33−4W¯5,  Φ¯10,11=2W¯5,Φ¯11,11=−2W¯4−2W¯5,  Φ¯11,12=2W¯4,Φ¯12,12=−S¯33−4W¯4,Y=(AP¯+DL000000BP¯0000)T,

and the rest of the items of (31) are all zero.

Furthermore, a desired controller gain matrix is given by

(36) $$\begin{array}{c} K=L{\left(\bar{P}\right)}^{-1}. \end{array}$$

**Proof**

When the controller *u*(*t*) = *Kx*(*t*), then the closed-loop system (1) is formulated as follows:

(37) $$\begin{array}{c} \dot{x}\left(t\right)=\left(A+DK\right)x\left(t\right)+Bx\left(t-d_{1}\left(t\right)-d_{2}\left(t\right)\right). \end{array}$$

Replace *A* with (*A* + *DK*) in Theorem 3, and use the Schur complement, (10) can be expressed as

(38) $$\begin{array}{c} \Phi +Y_{1}MY_{1}^{T}<0 \end{array}$$

with *Y*_1_ = (*A*+*DK* 0 0 0 0 0 0 *B* 0 0 0 0)^*T*^. Let block diagonal matrices *J*_1_ = diag⁡{*P* ^−1^,*P* ^−1^,…, *P* ^−1^} with 12 dimensions, *J*_2_ = diag⁡{*P* ^−1^,*P* ^−1^,*P* ^−1^}, and *J*_3_ = diag⁡{*P* ^−1^,*P* ^−1^}. And $\bar{P}=P^{-1}$, ${\bar{Z}}_{i}=P^{-1}Z_{i}P^{-1}\;(i=1,2,3)$, ${\bar{W}}_{i}=P^{-1}W_{i}P^{-1}\;(i=1,2,3)$, ${\bar{Q}}_{11}=P^{-1}Q_{11}P^{-1}$, ${\bar{Q}}_{12}=P^{-1}Q_{12}P^{-1}$, ${\bar{Q}}_{22}=P^{-1}Q_{22}P^{-1}$, ${\bar{R}}_{11}=P^{-1}R_{11}P^{-1}$, ${\bar{R}}_{12}=P^{-1}R_{12}P^{-1}$, ${\bar{R}}_{22}=P^{-1}R_{22}P^{-1}$, ${\bar{S}}_{ij}=P^{-1}S_{ij}P^{-1}\;(j>i$, *i* = 1,2,3,4, *j* ≤ 4), and ${\bar{T}}_{ij}=P^{-1}T_{ij}P^{-1}\;(j>i,\;i=1$,2,*j* ≤ 3). With these notations and (36) in mind, performing a congruence transformation to (38) and (11)-(12) by *J*_1_, *J*_2_, and *J*_3_, respectively, and by Schur complements, one can get

(39) $$\begin{array}{c} \left(\begin{array}{cccccc} \bar{\Phi } & Y & Y & Y & Y & Y\\ ★ & \Omega_{1} & 0 & 0 & 0 & 0\\ ★ & ★ & \Omega_{2} & 0 & 0 & 0\\ ★ & ★ & ★ & \Omega_{3} & 0 & 0\\ ★ & ★ & ★ & ★ & \Omega_{4} & 0\\ ★ & ★ & ★ & ★ & ★ & \Omega_{5} \end{array}\right)<0, \end{array}$$

(40) $$\begin{array}{c} \bar{S}=\left(\begin{array}{cccc} {\bar{S}}_{11} & {\bar{S}}_{12} & {\bar{S}}_{13} & {\bar{S}}_{14}\\ {\bar{S}}_{12}^{T} & {\bar{S}}_{22} & {\bar{S}}_{23} & {\bar{S}}_{24}\\ {\bar{S}}_{13}^{T} & {\bar{S}}_{23}^{T} & {\bar{S}}_{33} & {\bar{S}}_{34}\\ {\bar{S}}_{14}^{T} & {\bar{S}}_{24}^{T} & {\bar{S}}_{34}^{T} & {\bar{S}}_{44} \end{array}\right)>0, \end{array}$$

(41) $$\begin{array}{c} \bar{\Gamma }=\left(\begin{array}{ccc} {\bar{W}}_{2} & {\bar{T}}_{12} & {\bar{T}}_{13}\\ {\bar{T}}_{12}^{T} & {\bar{W}}_{2} & {\bar{T}}_{23}\\ {\bar{T}}_{13}^{T} & {\bar{T}}_{23}^{T} & {\bar{W}}_{2} \end{array}\right)\geq 0, \end{array}$$

with

(42) $$\begin{array}{c} \Omega_{1}=-d_{1}^{-2}{\bar{P}}^{-1}{\bar{W}}_{1}^{-1}{\bar{P}}^{-1},\;\;\Omega_{2}=-d_{2}^{-2}{\bar{P}}^{-1}{\bar{W}}_{2}^{-1}{\bar{P}}^{-1},\\ \Omega_{3}=-d^{-2}{\bar{P}}^{-1}{\bar{W}}_{3}^{-1}{\bar{P}}^{-1},\;\;\Omega_{4}=-d_{1}^{-2}{\bar{P}}^{-1}{\bar{W}}_{4}^{-1}{\bar{P}}^{-1},\\ \Omega_{5}=-d_{2}^{-2}{\bar{P}}^{-1}{\bar{W}}_{5}^{-1}{\bar{P}}^{-1}, \end{array}$$

where $\bar{\Phi }$ and *Y* are defined in Theorem 3. Noting that there exists a positive number *λ* such that

(43) $$\begin{array}{c} \left(\lambda \bar{P}-\bar{W_{i}}\right){\bar{W}}_{i}^{-1}\left(\lambda \bar{P}-{\bar{W}}_{i}\right)\geq 0\;\left(i=1,2,3\right), \end{array}$$

it is easy to see that

(44) $$\begin{array}{c} -\bar{P}{\bar{W}}_{i}^{-1}\bar{P}\leq -\frac{2}{\lambda }\bar{P}+\frac{1}{\lambda^{2}}{\bar{W}}_{i}\;\left(i=1,2,3\right). \end{array}$$

Therefore, (31)–(33) hold if (10)–(12) hold. The proof is completed.

**Remark 8**

It is very important to introduce a positive scalar *λ* to the effectiveness of Theorem 7. It is high load for −*P* to stabilize the five positive definite matrices *W*_*i*_  (*i* = 1,2,…, 5) stabilized in (39). Therefore, *λ* could play an important role in adjusting the computation of LMIs in Theorem 7.

## 4. Numerical Examples

In order to show the reduced conservatism and the effectiveness of the approaches presented in this paper, in this section, two numerical examples are provided.

**Example 9 (see [21])**

Consider the delayed system (6) with

(45) A=(−200−0.9),  B=(−10−1−1),d˙1(t)≤0.1,  d˙2(t)≤0.8.

We intend to find the upper bound *d*_2_ of *d*_2_(*t*) when *d*_1_ is known and the upper bound *d*_1_ of *d*_1_(*t*) when *d*_2_ is known, below which the system is asymptotically stable; see Table 1.

From Table 2, it is easy to see that our proposed stability criterion gives a less conservative result than the one in [16–21], when *d*_2_ is known, however, our condition is only less conservative than [16–20] when the *d*_1_ gets bigger and bigger, such as *d*_1_ = 1.2 and *d*_1_ = 1.5. So, this leaves much room for improvement on the stability conditions of dynamical systems with two additive time-varying delays. This should be a goal of future studies.

**Example 10 (see [21])**

Consider the dynamical system (5) with

(46) $$\begin{array}{c} A=\left(\begin{array}{cc} 2 & 0\\ 0 & 0.9 \end{array}\right),\;\;B=\left(\begin{array}{cc} -1 & 0\\ -1 & -1 \end{array}\right),\;\;D=\left(\begin{array}{cc} 1 & -2\\ -1.2 & 0.8 \end{array}\right),\\ {\dot{d}}_{1}\left(t\right)\leq 0.1,\;\;{\dot{d}}_{2}\left(t\right)\leq 0.8. \end{array}$$

If we set *d*_1_ = 1, *d*_2_ = 5.334, and *λ* = 1/500, it is clear to see that the systems cannot be stable without the controller. However, with the controller, the dynamical systems with two additive time-varying delays can be stabilized. By computing in the MATLAB, we can obtain some matrices as

(47) $$\begin{array}{c} \bar{P}=\left(\begin{array}{cc} 1.7347 & -0.7745\\ -0.7745 & 1.7318 \end{array}\right),\;\;\;L=\left(\begin{array}{cc} 3.8075 & 7.2104\\ 7.9126 & 2.8749 \end{array}\right),\\ K=L{\bar{P}}^{-1}=\left(\begin{array}{cc} 5.0657 & 6.4292\\ 6.6260 & 4.6236 \end{array}\right),\\ {\bar{W}}_{1}=\left(\begin{array}{cc} 0.0068 & -0.0031\\ -0.0031 & 0.0068 \end{array}\right),\;\;{\bar{W}}_{2}=\left(\begin{array}{cc} 0.0033 & -0.0027\\ -0.0027 & 0.0040 \end{array}\right),\\ {\bar{W}}_{3}=\left(\begin{array}{cc} 0.0022 & -0.0022\\ -0.0022 & 0.0029 \end{array}\right),\;\;{\bar{W}}_{4}=\left(\begin{array}{cc} 0.0068 & -0.0031\\ -0.0031 & 0.0068 \end{array}\right),\\ {\bar{W}}_{5}=\left(\begin{array}{cc} 0.0033 & -0.0026\\ -0.0026 & 0.0040 \end{array}\right),\;\;{\bar{Z}}_{1}=\left(\begin{array}{cc} 0.7096 & -0.0646\\ -0.0646 & 0.3778 \end{array}\right),\\ {\bar{Z}}_{2}=\left(\begin{array}{cc} 6.7190 & -0.1826\\ -0.1826 & 2.3906 \end{array}\right),\;\;{\bar{Z}}_{3}=\left(\begin{array}{cc} 0.7016 & -0.0470\\ -0.0470 & 0.3539 \end{array}\right). \end{array}$$

This example shows again that our approach is effective for the stabilization for the control system with two additive time-varying delays.

## 5. Conclusion

In this paper, we study the problem of stabilization for delay system with two additive time-varying delays. First of all, the less conservative delay-dependent stability conditions are given by constructing a new Lyapunov functional based on the ideal of delay decomposition, combining the analysis technique of inequalities with the reciprocally convex approach. And then, the controller of the closed-loop system is designed by the transformation technique of inequalities. Finally, two examples are analyzed to show the less conservative than some existing results and the effectiveness of our approach provided in this paper.

## Figures and Tables

**Table 1:** The maximal allowable bounds of *d*_1_ when *d*_2_ is known.

Method	*d*_1_	1	1.2	1.5
[16]	*d*_2_	0.415	0.376	0.248
[17]	*d*_2_	0.512	0.406	0.283
[18]	*d*_2_	0.872	0.672	0.371
[19]	*d*_2_	0.5188	0.4528	0.3777
[20]	*d*_2_	0.8731	0.6766	0.4529
[21]	*d*_2_	0.983	0.849	0.671

Theorem 3	*d*_2_	0.8731	0.6883	0.5381

**Table 2:** The maximal allowable bounds of *d*_2_ when *d*_1_ is known.

Method	*d*_2_	0.1	0.2	0.3
[16]	*d*_1_	2.263	1.696	1.324
[17]	*d*_1_	2.300	1.779	1.453
[18]	*d*_1_	1.770	1.672	1.571
[19]	*d*_1_	2.9182	2.3304	1.8324
[20]	*d*_1_	2.5583	2.1003	1.8083
[21]	*d*_1_	3.057	2.647	2.329

Theorem 3	*d*_1_	4.334	3.248	2.450
